# Experimental Investigation on Ambient-Cured One-Part Alkali-Activated Binders Using Combined High-Calcium Fly Ash (HCFA) and Ground Granulated Blast Furnace Slag (GGBS)

**DOI:** 10.3390/ma15041612

**Published:** 2022-02-21

**Authors:** Wee Teo, Kazutaka Shirai, Jee Hock Lim, Lynne B. Jack, Ehsan Nikbakht

**Affiliations:** 1 School of Energy, Geoscience, Infrastructure and Society (EGIS), Heriot Watt University Malaysia, Putrajaya 62200, Malaysia; 2 Faculty of Engineering, Hokkaido University, Sapporo 060-8628, Japan; shirai.kazutaka@eng.hokudai.ac.jp; 3 Department of Civil Engineering, Universiti Tunku Abdul Rahman, Bandar Sungai Long, Cheras, Kajang 43000, Malaysia; limjh@utar.edu.my; 4 School of Energy, Geoscience, Infrastructure and Society (EGIS), Heriot-Watt University, Edinburgh EH14 4AS, UK; l.b.jack@hw.ac.uk; 5 Department of Civil and Environmental Engineering, Universiti Teknologi Petronas, Seri Iskandar 32610, Malaysia; ehsan.nikbakht@utp.edu.my

**Keywords:** one-part, alkali-activated binder, high-calcium fly ash, ground granulated blast furnace slag

## Abstract

The challenges of handling user-hostile alkaline solutions in the conventional alkali-activated binders (AAB) have initiated the development of “just add water” or one-part solid-based AAB systems. This paper aims to present a preliminary investigation on the development of one-part ambient-cured alkali-activated binders produced by synthesising high-calcium fly ash (HCFA) and ground granulated blast furnace slag (GGBS) using sodium metasilicate anhydrous. Three test series were conducted in this study to investigate the effects of GGBS/binder, activator/binder and water/binder ratios on the fresh and hardened properties of the one-part synthesis AAB system. It was found that the SiO_2_/Al_2_O_3_ molar ratio plays an important role in the attainment of compressive strength and limits the amounts of solid activators effective in contributing to the alkali-activation reaction process. The optimum SiO_2_/Al_2_O_3_ molar ratio was found between 3.20 and 3.30. The test results revealed that the optimum proportion between HCFA and GGBS was discovered at a GGBS/binder ratio of 0.50. The optimum activator/binder ratio was between 0.08 and 0.12, and it is recommended that the water/binder ratio should not exceed 0.50. This study demonstrated the potential of the one-part synthesis method in the production of alkali-activated binder for practical structural applications.

## 1. Introduction

Ordinary Portland Cement (OPC) is one of the most widely used construction materials worldwide. It is strong, durable, cheap, and abundant in raw materials all over the world. OPC manufacturing processes are known to be highly energy-intensive, which releases huge amounts of carbon dioxide (CO_2_) emissions into the environment. It is generally known that for every tonne of OPC, almost one tonne of CO_2_ is emitted. Given that the cement industry is responsible for up to 7% of the world’s carbon emissions [1], there is an urgent driving need to develop more technically viable solutions or alternatives to conventional OPC with minimised energy consumption and carbon emissions.

Alkali-activated binder (AAB) is considered one of the promising contenders as a viable substitute for OPC. This binder is produced by synthesising aluminosilicate source materials of geological origin or industrial by-products with highly alkaline activators. The most commonly used aluminosilicate sources for alkali activation are fly ash (FA), ground granulated blast furnace slag (GGBS), metakaolin (MK), rice husk ash (RHA) and palm oil fuel ash (POFA) [2,3,4,5,6,7]. AAB has been regarded as an important element of “sustainable cementing binder systems” for the future [8,9]. It offers a potential reduction in CO_2_ emissions by as much as 80% or more, when compared to OPC [10]. Due to its great potential, over the past decades, there has been growing interest in the development and application of AAB. This is clearly reflected by the large number of research articles published in various state-of-the-art reports [8,11,12,13,14].

There are basically two methods of producing AAB, either a one-part mix or a two-part mix formulation. Most of the research on AAB is primarily focused on the two-part mix type, also known as a solution-activated mixture. This type of AAB can be produced by synthesising aluminosilicate sources with highly concentrated alkaline solutions made up of sodium hydroxide (NaOH) and sodium silicate (Na_2_SiO_3_). Both NaOH and Na_2_SiO_3_ are known for being hazardous and highly corrosive and handling such user-hostile solutions can be extremely challenging, especially in large quantities for commercial applications. One-part or “just add water” AAB is developed specially with the intention of tackling the challenges of handling solution-based alkaline activators. Both aluminosilicate sources and alkaline activators are in solid form (powder-based) and then blended to make the one-part binder system. The activation process begins once water is added to the solid mixture. The whole mixing and handling process is thus similar to conventional OPC concrete. The use of the one-part system could increase the commercial viability of AAB for mass production and large-scale applications in the construction industry.

Over the past decades, there have been several attempts at the synthesis of one-part AAB with various combinations of aluminosilicate sources and solid activators. Yang et al. [15] and Yang and Song [16] developed cementless binders using FA and GGBS as the source materials and activated by either sodium silicate powder or a combination of sodium silicate and sodium hydroxide powders. GGBS-based AAB achieved higher compressive strength than FA-based AAB, with more notable strength development at an early age. The FA-based AAB they produced showed very low 28-day compressive strengths, in some cases less than 1 MPa. Hajimohammadi et al. [17] successfully produced a one-part geopolymer system utilising geothermal silica and solid sodium aluminate. They found that samples with less water content had a greater extent of crystallite formation, possibly due to the rapid dissolution of the aluminate source and the relatively slower dissolution of the silica source in the early stages of geopolymerisation. Hajimohammadi and van Deventer [18] studied the effects of water content and Si/Al ratio on the reaction mechanisms and physical properties of fly ash one-part systems synthesised by solid sodium silicate. They found that the rates of release of Si and Al nutrients from source materials greatly affect their availability for reaction and their extent of participation in a geopolymer gel structure. In one-part mix systems, the usual crystalline phases that appear in fly ash geopolymers are missing, and by increasing the Si/Al ratio, the extent of Si contribution in the final geopolymer gel is reduced. Participation of more Si in the stages of gel formation can be anticipated for samples with a lower water content. Waste valorisation of FA and red mud to produce a one-part mix AAB was investigated by Choo et al. [19]. They found that one-part alkali-activated FA could be synthesised at ambient temperatures using red mud as the solid alkali activator. However, the resulting compressive strengths were low, less than 2 MPa. This is possibly due to the low pH level (<11) of the materials they produced, which would have hindered the active polycondensation reaction. Kim et al. [20] found that calcium oxide (CaO) is a more effective activator for GGBS than calcium hydroxide (Ca(OH)_2_) in terms of achieving higher compressive strengths and producing more C-S-H gels.

Synthetic alkali silicates are the most widely used activators in either one-part or two-part AAB mixtures [21]. For one-part AAB, synthetic sodium metasilicate anhydrous has been proven to be highly effective for strength development [22,23,24]. Ma et al. [24] investigated three different types of commercial sodium metasilicate powders, namely Na_2_SiO_3_-anhydrous, Na_2_SiO_3_.5H_2_O and Na_2_SiO_3_.9H_2_O, respectively. In terms of strength development, Na_2_SiO_3_-anhydrous achieved the highest followed by Na_2_SiO_3_·9H_2_O, and Na_2_SiO_3_·5H_2_O was the lowest. The N-A-S-H gels developed in Na_2_SiO_3_-anhydrous and Na_2_SiO_3_·9H_2_O-activated geopolymers appear to be better geopolymerised compared to Na_2_SiO_3_·5H_2_O-activated geopolymers. To further minimise the CO_2_ emissions, alkalinity and cost associated with Na_2_SiO_3_-anhydrous activated geopolymers, Ma et al. [25] explored the possibility of partially replacing a portion of Na_2_SiO_3_-anhydrous with sodium carbonate (Na_2_CO_3_). The results showed that increasing the Na_2_CO_3_ content decreases the compressive strength and the quantity of gelatinous products, which eventually led to many large micro-cracks and high porosity. Although a combined composite activator is an attractive and cleaner solution, somehow this may sacrifice the performance and economic benefit. To improve the commercial practicality of geopolymers, Askarian et al. [26] studied various types of solid activators, such as sodium silicate, calcium hydroxide, sodium oxide, lithium hydroxide, potassium carbonate and their combinations. They found that under the ambient-cured condition, the highest compressive strength was obtained from a precursor made up of 50% fly ash and 50% slag activated by a combination of sodium silicate, calcium hydroxide and lithium hydroxide.

Regarding the precursor materials, the majority of the studies used low-calcium FA as the aluminosilicate source. The use of high-calcium FA for one-part AAB systems is extremely rare due to its flash setting and low workability characteristics [27]. In this study, high-calcium FA combined with GGBS is synthesised using sodium metasilicate anhydrous to produce an ambient-cured one-part AAB system. The use of GGBS enables to eliminate the necessity of heat curing [5]. The effect of GGBS/binder, solid activator/binder and water/binder ratios on the fresh and hardened properties of one-part AAB systems are thoroughly investigated in this paper. Optimum proportion ratios as well as their statistical correlations are proposed to pave the way for one-part AAB technology to be deployed in future construction practices.

## 2. Experimental Programme

### 2.1. Materials

#### 2.1.1. High-Calcium Fly Ash (HCFA)

Fly ash (FA) used in this study was obtained locally from YTL Cement Bhd Malaysia. The oxide analysis of the FA determined by X-ray fluorescence (XRF) analysis is shown in Table 1. The pozzolanic compounds level of the FA used in this study (SiO_2_ + Al_2_O_3_ + Fe_2_O_3_) is 61.9% and can therefore be classified as Class C fly ash according to ASTM C618-12a [28] (i.e., >50%). The FA contains a relatively high percentage of CaO (18.94%), greater than 10%, and therefore is classified as high-calcium fly ash (HCFA).

#### 2.1.2. Ground Granulated Blast Furnace Slag (GGBS)

Ground granulated blast furnace slag (GGBS) used in this study was obtained from the same supplier (YTL Cement Bhd Malaysia). The oxide composition of the GGBS is shown in Table 1. It was off-white in colour and contained at least two-thirds by mass of glassy slag and possesses hydraulic properties when suitably activated. The sum of CaO, MgO and SiO_2_ in the GGBS constituted 86.8% of the total mass, while the basicity ratio (CaO + MgO)/(SiO_2_) was 1.56, both of which satisfy the requirements specified in BS EN 197-1: 2011 [29] (i.e., two-thirds for the sum of CaO, MgO and SiO_2_ (by mass) and >1.0 for the basicity ratio).

#### 2.1.3. Solid Activator

One-part or “just add water” geopolymers correspond to using a solid-based alkaline activator to synthesise geopolymer composites. The solid alkali activator used in this study was sodium metasilicate anhydrous, which is a similar solution to that adopted by Nematollahi et al. [22]. The chemical contents of the sodium metasilicate anhydrous used in this study were 51.10% Na_2_O and 46.53% SiO_2_, with a modulus ratio (Ms) and relative density of 0.91 and 2.4, respectively.

### 2.2. Design Mix Proportions

The main purpose of this research is to investigate the main influencing parameters in the development of appropriate mix proportions of an ambient-cured one-part alkali-activated paste. The mixture consists of four main ingredients: high-calcium fly ash (HCFA), ground granulated blast furnace slag (GGBS), solid activator (sodium metasilicates anhydrous) and water. Three test series were planned in sequence to investigate the three main influencing parameters, namely the ratios of GGBS/binder, activator/binder and water/binder, summarised as follows:

1. Series A: This test series was the first series conducted to define the appropriate binder proportion between HCFA and GGBS. The GGBS/binder ratio was taken as the main parameter in this investigation. The activator/binder and water/binder ratios were kept constant in this series at 0.12 and 0.40, respectively. A total of 8 mixes with various proportions between HCFA and GGBS were conducted.
2. Series B: This test series was intended to evaluate the effect of the activator/binder ratio on the one-part alkali-activated paste. After the evaluation of Series A, the optimum GGBS/binder ratio will be used in this series. Additionally, the water/binder ratio was kept constant at 0.40. In total, there are 6 mixes in this series.
3. Series C: In this final test series, the main variable will be the water/binder ratio. In total, there are 4 mixes in this series. The GGBS/binder ratio and activator/binder ratio used are based on the results obtained from Series A and B, respectively.

According to Yang et al. [15,16], the SiO_2_/Al_2_O_3_ molar ratio and calcium content (CaO) in the source material, and the Na_2_O/SiO_2_ molar ratio in the alkaline activator, play very important roles on the alkali-activation mechanisms and mechanical properties of the one-part geopolymer pastes. Recognising the importance, the above-proposed test series will also include those molar ratios in their investigation. The effect of SiO_2_/Al_2_O_3_, Na_2_O/SiO_2_ and H_2_O/Na_2_O molar ratios will form part of the main study in test series A, B and C, respectively.

### 2.3. Mixing, Casting and Curing

All mixes were prepared by using a 5-litre planetary mixer. Solid and dry materials, which are comprised of FA, GGBS and solid activator, were first added into the mixer and then dry-mixed for 3 min. The water was then gradually added to the dry materials and the mixing continued for a further 3 min to produce the fresh and uniform paste. The fresh paste was then cast into 50 mm cube moulds for the compressive strength test.

Immediately after casting, the moulds were covered with plastic film to avoid water evaporation and then left overnight for demoulding the following day. After being demoulded, the specimens were fully wrapped with a plastic sheet and left to cure under ambient conditions in the laboratory until the day of testing.

### 2.4. Test Methods

Two test methods were adopted in this experiment programme. Firstly, the flow table test (or flow test) as per the standard ASTM C230 [30] was conducted immediately after mixing to determine the consistency of the fresh geopolymer paste. The target flow spread diameter was aimed for a minimum of 200 mm so that desirable rheological properties and self-compacting capability of the paste can be achieved. Secondly, compressive strength tests were conducted at 3, 7, 14 and 28 days of age. A total of twelve 50 mm cube moulds were cast in each mixture to determine the compressive strength. Prior to testing, each specimen was weighed to obtain their respective densities.

## 3. Results and Discussion

### 3.1. Test Series A: Proportion of HCFA and GGBS

As mentioned in the preceding section, test series A consisted of 8 mixes where the primary variable is the binder proportion between HCFA and GGBS or the GGBS/binder ratio. The activator/binder and water/binder ratios were kept constant at 0.12 and 0.40, respectively. Table 2 summarises the mixture proportions and the molar ratios of critical chemical compounds in the mixtures for this test series.

#### 3.1.1. Fresh Paste Properties

**Figure 1:**
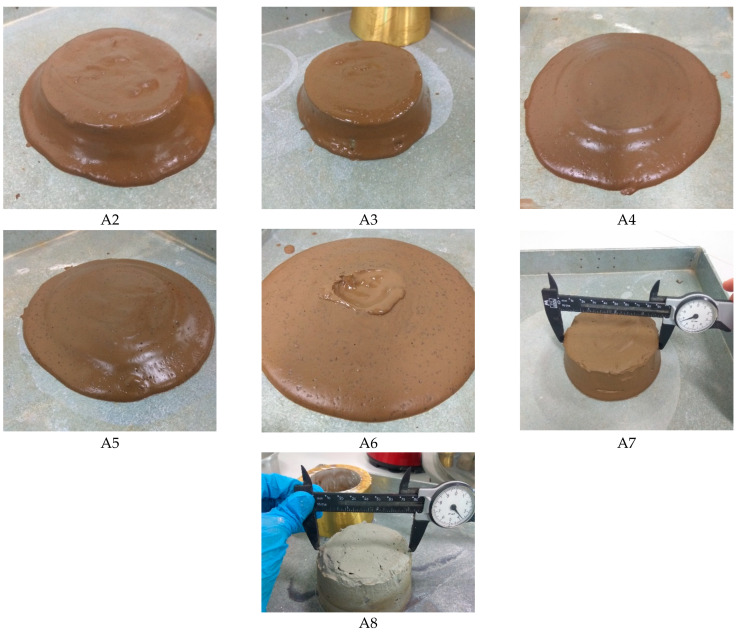
Flow characteristic from test series A.

Figure 1 shows the flow characteristics of each fresh mixture immediately after mixing (except for A1). Based on observation, the flow characteristics of mixtures A1, A2 and A3 form a thick and highly cohesive paste. The fresh mixture tends to flow by gravity and shows a viscous property. Due to the highly cohesive paste, no flow spread diameter readings were taken for those mixtures. The observed fresh properties’ behaviour was mainly due to the low reactivity of HCFA. When activated with a solid activator, the HCFA remained either unreactive or partially reactive. Sasui et al. [31] revealed that low reactivity of Class C FA limits the leaching of major elements such as Si, Al and Ca to the matrix, which could prolong the setting time and limit the formation of calcium silicate hydrate (C-S-H) gel for strength development.

**Figure 2:**
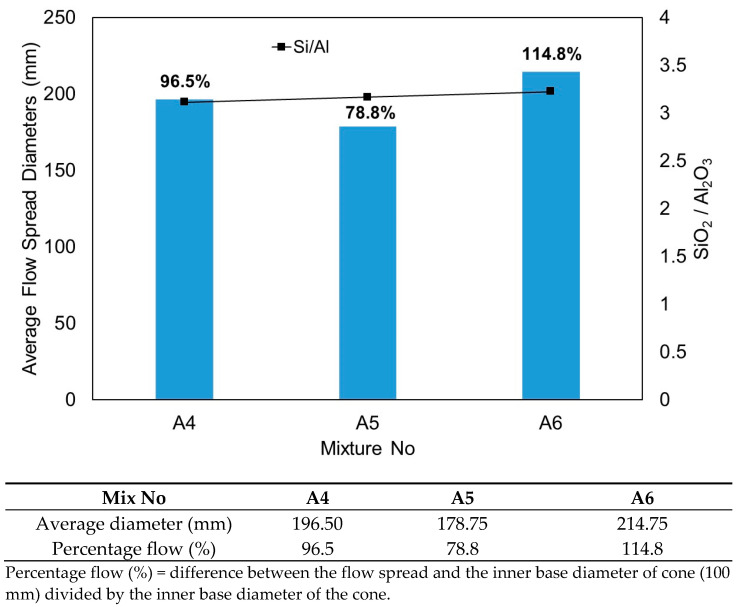
Correlation of SiO_2_/Al_2_O_3_ molarity ratio and average flow spread diameters taken from mixtures A4, A5 and A6.

With a further increase in the replacement level of GGBS, there was a dramatic change in the flowability of the mixture. As shown in Figure 1, mixtures A4, A5 and A6 exhibited better flowability spread than mixtures A2 and A3. The results of flow table tests taken from mixtures A4, A5 and A6 are plotted in Figure 2. Also included in the figure are the percentage flow and the variation of the SiO_2_/Al_2_O_3_ molar ratio.

GGBS is more reactive than FA in improving flowability. The results show that an increase in the replacement level of HCFA by GGBS between 30% and 50% dramatically improved the flowability of the mixtures. Mixture A4 with a 30% replacement level of GGBS (GGBS/binder = 0.30) obtained an average flow spread diameter of 196.5 mm, which corresponds to 96.5% flow. The highest average flow spread diameter of 214.75 mm (114.8% flow) was achieved with mixture A6, with a 50% replacement level (GGBS/binder = 0.50). This replacement level is considered to be the threshold level. As observed from Figure 1, when GGBS exceeded 50% (A7 and A8), the mixture became stiff with a quick setting time. Similar observations were also reported by other researchers [5,32] on the effect of an increase of GGBS content with a decrease in the initial and final setting times.

#### 3.1.2. Density

**Figure 3:**
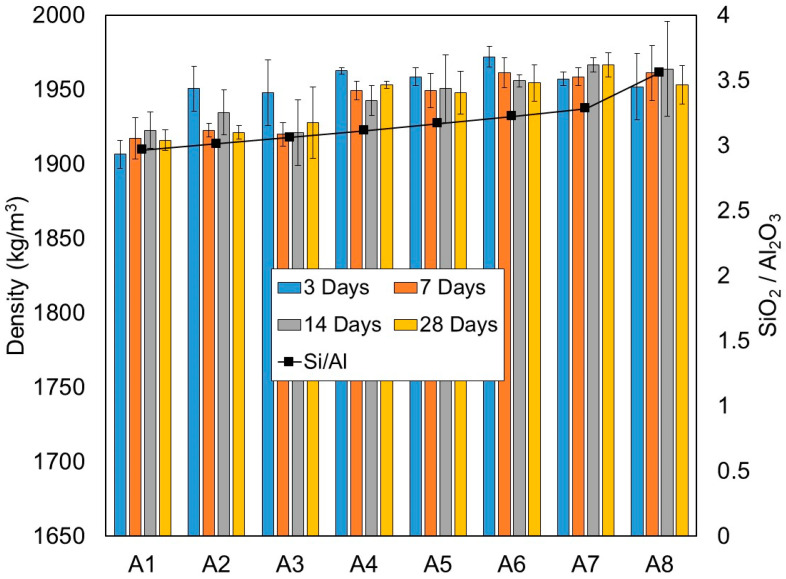
Density of one-part alkali-activated pastes from test series A (kg/m^3^), at the 3, 7, 14 and 28 days curing periods.

The dry density of one-part alkali-activated paste in each mixture was measured at 3, 7, 14 and 28 days. All results are presented in Table 3 and Figure 3. The density was evaluated by weighing the mass and dimensions of three 50 mm cubes per age and taking the average value.

Generally, dry density increases in relation to the SiO_2_/Al_2_O_3_ molar ratio and replacement level of GGBS. As can be seen from Figure 3, the densities increased along with the steady increase of the SiO_2_/Al_2_O_3_ molar ratio due to the increase in the replacement level of GGBS or the GGBS/binder ratio. It is evident that the increases in densities were more pronounced when the GGBS/binder ratio exceeded 0.30. On average, the dry densities achieved 1955 kg/m^3^ at 28 days. It is interesting to note that mixture A8 with a GGBS/binder ratio of 1.0, despite having the highest SiO_2_/Al_2_O_3_ molar ratio (3.56), did not exhibited the highest densities. As a matter of fact, it had lower densities at 28 days compared to mixture A7.

Based on the observation, mixtures with combined HCFA/GGBS (such as mixtures A2–A6) demonstrated the highest rates of dry density gain at an early age of 3 days, with densities ranging from 1951 to 1972 kg/m^3^. A slight decrease in density could be observed at later ages, typically around a 0.5–1.5% reduction. This phenomenon is probably due to the low alkali-activation reaction and water evaporation at later ages of curing.

#### 3.1.3. Compressive Strength

**Figure 4:**
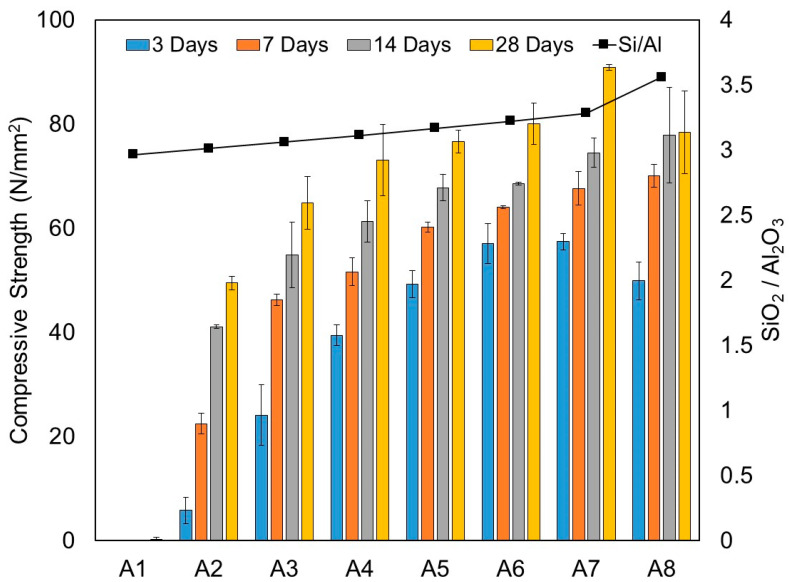
Correlation of SiO_2_/Al_2_O_3_ molar ratio and compressive strength at 3, 7, 14 and 28 days of one-part alkali-activated pastes from test series A (N/mm^2^).

The results of the compressive strength obtained at 3, 7, 14 and 28 days with all mixtures are presented in Table 4 and Figure 4. It should be noted that setting and hardening of mixture A1 with 100% HCFA did not occur satisfactorily and strength development failed to take place throughout the whole age of curing. As mentioned in Section 3.1.1, this is mainly due to the low reactivity of HCFA that limits the leaching of Ca to the matrix. As mentioned by Luukkonen et al. [33], the composition of binding phases of alkali-activated binders is greatly affected by the Ca content: N-A-S-H, C-(N)-A-S-H and C-A-S-H gels are formed in low-, intermediate-, and high-calcium systems, respectively. Yang et al. [16] and Kim et al. [20] observed that in high-calcium one-part systems, the main binding phase is likely to be crystalline C-S-H and also C-A-S-H gels. It is well-documented [31,34] that the formation of C-S-H/C-A-S-H gels produces a compact matrix and improves the strength.

It is obvious from Figure 4 that with GGBS introduced into the mixture, the gain of compressive strength significantly developed. As can be seen from the result, strength gain was directly proportional to the replacement levels of GGBS and the SiO_2_/Al_2_O_3_ molar ratio. The higher the content of GGBS, the greater the compressive strength development. Table 4 also includes the strength gain ratio, taken as the ratio of compressive strength at a designated age to the compressive strength at 28 days. It is noteworthy that early-age strength development was significant in the first 7 days. The compressive strength could reach up to 70–80% of the compressive strength at 28 days depending on the replacement level of GGBS. As shown in Table 4, the strength gain attainment at 3 and 7 days for mixture A6 with a GGBS/binder ratio of 0.50 was 71% and 80%, respectively. It is interesting to note that at 14 days, the strength attainment was shown as fairly constant between mixtures A2 and A7, regardless of the GGBS replacement level, with an average of 84.7% of compressive strength attained at 28 days.

Mixture A8 with 100% GGBS showed a lower compressive strength at an early age of 3 days and subsequently gained strength, with a higher compressive strength at 7 and 14 days compared to mixtures A6 and A7. It is important to note that at 14 days, the compressive strength actually attained almost its full strength of 78.45 MPa at 28 days. Despite the fact that mixture A8 had the highest SiO_2_/Al_2_O_3_ molar ratio (3.56), its compressive strength at 28 days was actually lower than mixtures A6 and A7. The results showed that at 28 days, mixtures A6 and A7 achieved compressive strengths of 80.1 and 90.9 MPa, respectively. This is an interesting observation and in line with findings reported by other researchers [18,27]. According to Chindaprasirt et al. [27], increased SiO_2_ content would lead to a decrease in compressive strength. They suggested that the optimum SiO_2_/Al_2_O_3_ molar ratio should fall between 3.20 and 3.70. Based on our test observations from mixtures A6, A7 and A8, our optimum SiO_2_/Al_2_O_3_ molar ratio was around 3.20 to 3.30. Once it passed the limit, a decrease in compressive strength could be observed. As depicted in mixture A8, with a SiO_2_/Al_2_O_3_ molar ratio equal to 3.56, it demonstrated a lower compressive strength at 28 days compared to mixtures A6 and A7.

#### 3.1.4. Discussion of Results from Test Series A

The GGBS/binder ratio was investigated in this series. FA used in this study is a Class C-type high-calcium (Ca) fly ash (HCFA). Despite the high Ca content, its reactivity is found to be either unreactive or partially reactive. Ca content has long been known for its rapid setting property, and hence low workability, and through the coexistence of C-S-H phase it improves the mechanical properties of the final products [27]. However, this does not fully reflect in the results obtained from this series. Although the flow characteristic of mixture A1 in fresh conditions exhibited a thick and highly cohesive paste, there was no sign of setting and hardening of the paste after 28 days of curing. As a matter of fact, the compressive strength development of mixture A1 failed to take place at all.

The addition of GGBS into the proportion improved the reactivity of the precursor material and enhanced the early strength development at ambient curing conditions. The flowability performed well between GGBS/binder ratios of 0.30 and 0.50. When the ratio exceeded 0.50, the flowability and setting time dramatically reduced, as shown in mixtures A7 and A8. Obviously, compressive strength development increases along with the GGBS content. An optimum balance between flowability in fresh conditions and compressive strength is required in order to achieve a feasible mixture. Based on the results from this series, the optimum proportion between HCFA and GGBS was found at a GGBS/binder ratio of 0.50. This was deduced based on the results of flow spread diameter and compressive strength from mixture A6. It achieved a compressive strength of 80 MPa at 28 days with a flow spread diameter of 214.75 mm (114.8% flow).

**Figure 5:**
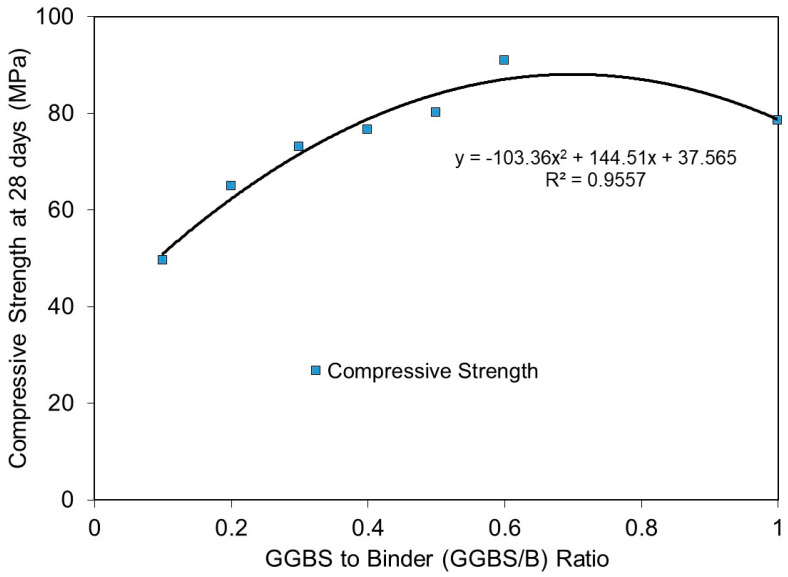
Relationship of compressive strength at 28 days with GGBS to binder ratios (GGBS/B ratio).

The relationship of compressive strength at 28 days with respect to GGBS/binder ratios is plotted in Figure 5. Generally, the results showed good correlations between compressive strength and GGBS/binder ratios, with a calculated correlation coefficient (R2) of 0.956. The trend showed that compressive strength increased with the GGBS/binder ratio, reached a maximum at a GGBS/binder ratio of approximately 0.70, which corresponds to a SiO_2_/Al_2_O_3_ molar ratio of 3.348, and then decreased afterward. It is noteworthy that the predicted maximum compressive strength at a SiO_2_/Al2O_3_ molar ratio of 3.348 corresponds well to our test observations, which found that the optimum SiO_2_/Al_2_O_3_ molar ratios were close, in the range between 3.20 and 3.30.

### 3.2. Test Series B: Activator to Binder Ratio

The main objective of test series B was to evaluate the optimum activator/binder (A/B) ratio. The GGBS/binder ratio was kept at 0.50 following the results obtained from test series A. The water/binder ratio was kept constant at 0.40. Table 5 summarises the mixture proportions and the molar ratios of critical chemical compounds in the mixtures for this test series. A total of six mixes including mixture A6 taken from test series A are listed in Table 5.

#### 3.2.1. Fresh Paste Properties

**Figure 6:**
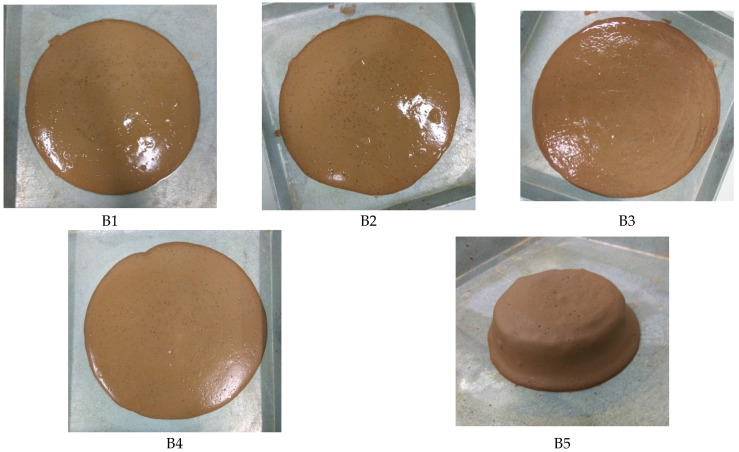
Flow characteristics from test series B.

**Figure 7:**
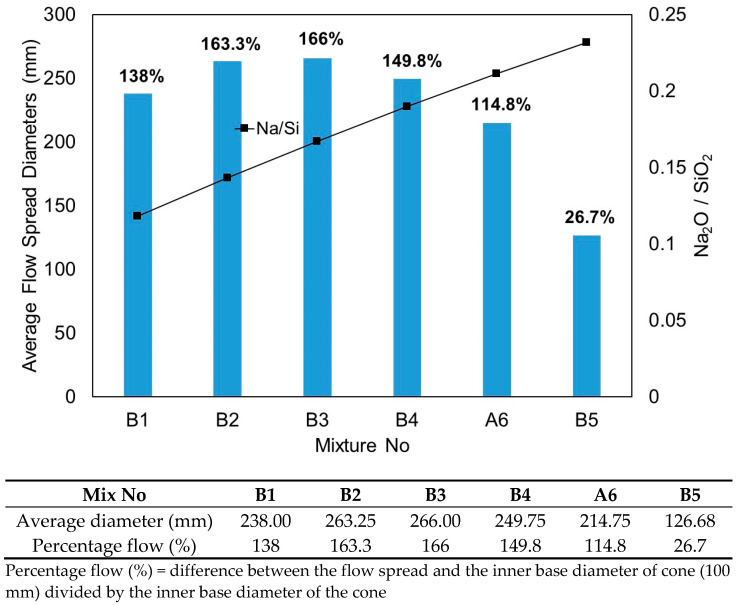
Correlation of Na_2_O/SiO_2_ molarity ratio and average flow spread diameters from test series B.

The flow characteristics of all mixtures in test series B are presented in Figure 6 and Figure 7. The flowability of the fresh state has been visually observed on all mixtures. As a general trend, all mixtures, except mixture B5, exhibited considerable flowability spread, with average flow spread diameters exceeding 200 mm. Based on the observation, mixture B5 displayed a highly cohesive and viscous paste with a fast setting time. The flow characteristics of the mixtures were greatly influenced by the Na_2_O concentration in the solid activators and source materials, which were commonly denoted by the Na_2_O/SiO_2_ molar ratio. Table 5 summarises the Na_2_O/SiO_2_ molar ratios of the mixtures in this test series. Figure 7 shows the relationship between average flow spread diameters and Na_2_O/SiO_2_ molar ratios of the mixtures.

From Figure 7, it is evident that initially, flowability increased with the increase of the Na_2_O/SiO_2_ molar ratio between 0.118 and 0.167, which corresponded to mixtures B1–B3 with activator/binder ratios of 0.04 to 0.08. Mixtures B2 and B3 achieved the highest average flow spread diameter with comparatively close values at 263.25 and 266 mm, respectively. Further increasing the Na_2_O/SiO_2_ molar ratio beyond 0.167 led to a decline in flowability, which implied that any higher amount of Na_2_O exceeding a Na_2_O/SiO_2_ molar ratio of 0.167 in the solid activators can significantly reduce the flowability of fresh pastes. The results revealed that mixture B3 with an activator/binder ratio of 0.08 (corresponds to a Na_2_O/SiO_2_ molar ratio = 0.167) proved to be the optimum amount of solid activator in terms of flowability. Interestingly, a similar finding was also reported by Oderji et al. [35].

#### 3.2.2. Density

**Figure 8:**
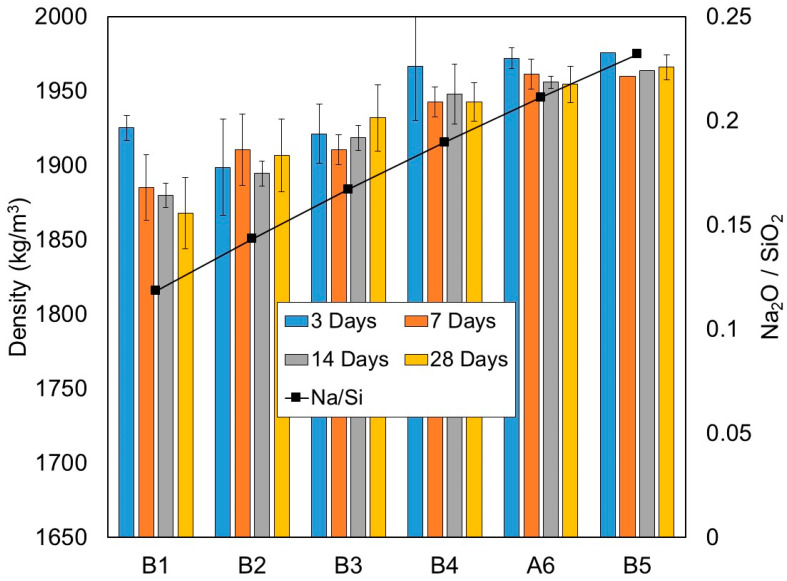
Density of one-part alkali-activated pastes from test series B (kg/m^3^), at the 3, 7, 14 and 28 days curing periods.

The dry densities of the one-part geopolymer paste in each mixture measured at 3, 7, 14 and 28 days are presented in Table 6 and Figure 8. The density was evaluated by weighing the mass and dimensions of three 50 mm cubes per age and taking the average value. For mixture B5, only one cube was prepared for ages of 3, 7 and 14 days due to the damage of specimens during the demoulding process.

Figure 8 shows that at all ages, the dry density increased with the increase in the amounts of solid activators or the Na_2_O/SiO_2_ molar ratio. The main reaction product in high-calcium one-part systems is likely crystalline C-S-H and also C-A-S-H gels. It is generally understood that a high Na_2_O/SiO_2_ molar ratio (or Na_2_O concentration) promotes high levels of the alkali-activation process, which leads to the formation of more C-S-H/C-A-S-H gels and a denser morphology [3,36,37].

Generally, the rate of dry density gain was demonstrated to be the highest at the early age of 3 days, with densities ranging between 1899 and 1976 kg/m^3^, and then it decreased at later ages, with the highest reduction up to 3.0%. These observations were clearly revealed in mixtures B1, B4, A6 and B5. The reduction is probably due to the low alkali-activation reaction and water evaporation at a later age of curing.

#### 3.2.3. Compressive Strength

**Figure 9:**
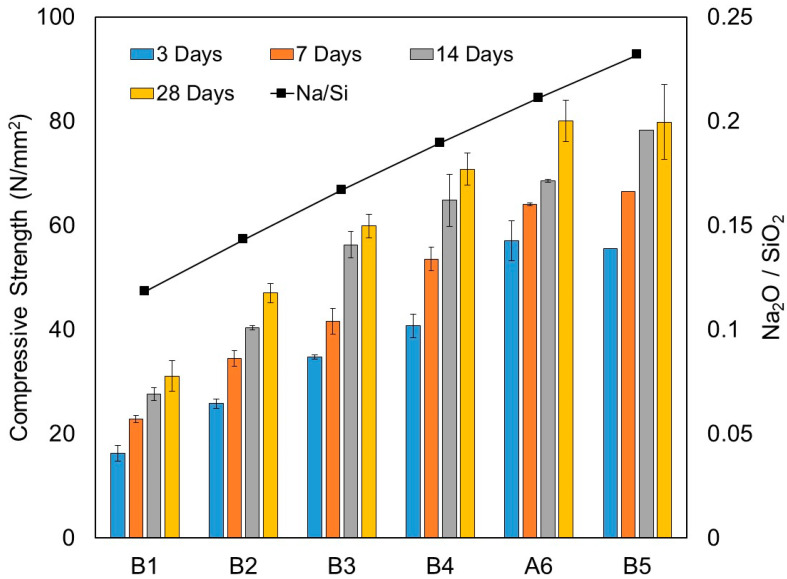
Correlation of Na_2_O/SiO_2_ molar ratio and compressive strength at 3, 7, 14 and 28 days of one-part alkali-activated pastes from test series B (N/mm^2^).

The results of compressive strength for test series B measured at 3, 7, 14 and 28 days are presented in Table 7 and Figure 9. Similar to dry densities, the compressive strength increased with the amount of solid activators or the activator/binder ratios. Figure 9 also shows the relationship between compressive strengths and the Na_2_O/SiO_2_ molar ratio.

Mixture B1 with an activator/binder ratio of 0.04 achieved a compressive strength of 31.1 MPa at 28 days. As shown in mixtures B2, B3 and B4, the compressive strength at 28 days increased by 51.3%, 92.6% and 127.8%, respectively, compared to mixture B1. The highest compressive strength at 28 days was attained from mixture A6 with an activator/binder ratio of 0.12 at the value of 80.1 MPa, which corresponds to a 157.5% increase compared to mixture B1. It should be noted that there was no sign of further increments in compressive strength in mixture B5 when the activator/binder ratio increased to 0.14. As a matter of fact, mixture B5 achieved an almost identical compressive strength value to mixture A6, of 79.8 MPa, at 28 days.

Table 7 presents the strength gain ratios calculated at 3, 7 and 14 days for all mixtures. In terms of compressive strength development, all mixtures could gain at least 50% and 70% of the compressive strength at 28 days at the early age of 3 and 7 days, respectively. The activator/binder ratio had a significant effect on the early-age compressive strength development. As demonstrated in mixtures A6 and B5, a higher activator/binder ratio resulted in higher strength gain ratios at 3 and 7 days compared to mixture B1. After 7 days, the strength gain appeared to be more consistent throughout all mixtures. It is interesting to note that at 14 days, mixture B5 attained almost equal to its full compressive strength at 28 days, with a strength gain ratio of 0.98. Additionally, as mentioned earlier, mixture B5 did not indicate any sign of further increments in compressive strength at 28 days and achieved an almost identical strength value to mixture A6. This observation implied that there is probably a limit on the effective amounts of solid activators needed to contribute to the alkali-activation reaction process.

The GGBS/binder ratio of the source material was kept constant in this series at 0.50. The SiO_2_/Al_2_O_3_ molar ratio of the mixtures increased due to the increase of the amount of Na_2_SiO_3_ solid activators. Mixture B5 had a SiO_2_/Al_2_O_3_ molar ratio equal to 3.30. As explained in Section 3.1.3, according to Chindaprasirt et al. [27], there is a limit on the extent of SiO_2_ content in relation to compressive strength. Once past the limit, it would lead to a decrease in compressive strength. Our findings from test series A suggested that our optimum SiO_2_/Al_2_O_3_ molar ratio fell between 3.20 and 3.30, which somehow explained why mixture B5 exhibited no further increase in strength.

According to Hajimohammadi et al. [18], a higher SiO_2_/Al_2_O_3_ (Si/Al) molar ratio could potentially decrease the amount of Si contribution in the final geopolymer gel and the geopolymerisation reaction rate in FA one-part geopolymers. This is mainly due to the formation of larger unreactive silica oligomers instead of small reactive species at a high Si/Al ratio. From their microstructural analysis, it showed that for samples with a high Si/Al ratio, the microstructure was less dense with larger pore formation. In terms of mechanical strength, they also indicated lower compressive strengths with increased Si/Al ratios.

#### 3.2.4. Discussion of Results from Test Series B

This test series investigated the influence of the solid activator/binder (A/B) ratio on the fresh and mechanical properties of an ambient-cured one-part alkali-activated binder. Sodium metasilicate (Na_2_SiO_3_) anhydrous was used as the solid alkaline activator. Often, the activator concentration is expressed by the Na_2_O/SiO_2_ molar ratio. It is well-documented that the Na_2_O/SiO_2_ molar ratio plays a vital role in the alkali-activation process [3,36,37,38].

High Na_2_O/SiO_2_ molar ratios encouraged more alkali activation with the aluminosilicate materials (in this case, combined HCFA/GGBS) and led to the formation of more C-S-H/C-A-S-H gel, denser morphology, lower porosity and therefore higher compressive strength. As demonstrated from our results, dry densities and compressive strength increased with the Na_2_O/SiO_2_ molar ratio. However, there appeared to be a limit on the effective amounts of solid activators in the contribution to the alkali-activation reaction process. Our results indicated that this limit is greatly influenced by the SiO_2_/Al_2_O_3_ molar ratio. According to Section 3.1, the optimum SiO_2_/Al_2_O_3_ molar ratio should fall between 3.20 and 3.30. Any mixtures exceeding this range of the SiO_2_/Al_2_O_3_ molar ratio would not show further increases in compressive strength, even when provided with high amounts of solid activators, as clearly demonstrated in mixture B5 in this series.

**Figure 10:**
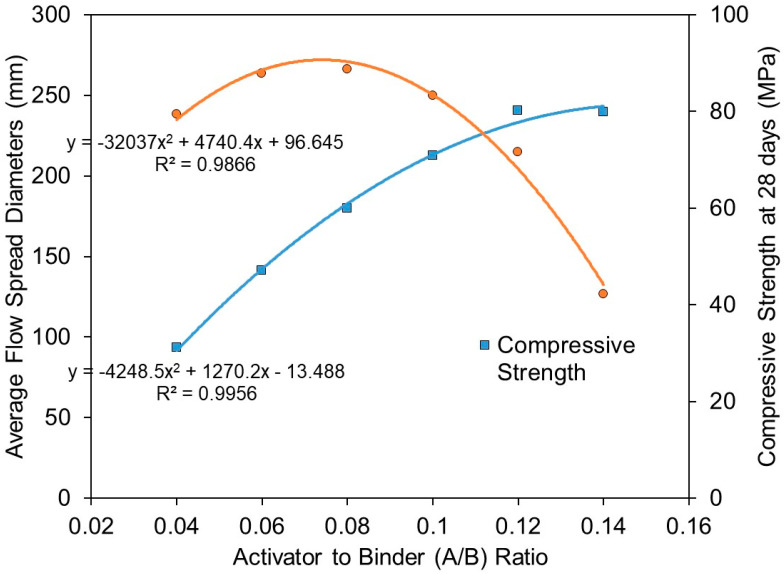
Relationship of average flow spread diameters and compressive strength at 28 days with activator to binder ratios (A/B ratio).

The relationship of flowability (in terms of average flow spread diameters) and compressive strength at 28 days with respect to activator/binder ratios is plotted in Figure 10. It is evident that flowability and compressive strength showed good correlations with activator/binder ratios. The calculated correlation coefficients (R2) for flowability and compressive strength were 0.987 and 0.996, respectively. Both predicted trends follow closely to the actual test results. The results showed that mixtures with an activator/binder ratio beyond 0.12 (such as mixture B5, activator/binder ratio = 0.14, SiO_2_/Al_2_O_3_ ratio = 3.301) did not reveal a noticeable improvement in compressive strength. Whereas in terms of flowability, a pronounced effect in reducing the flowability of the mixture could be observed after the activator/binder ratio exceeded 0.08.

Given the above interpretation, it could be deduced that activator/binder ratios in the range between 0.08 and 0.12, which corresponds to a Na_2_O/SiO_2_ molar ratio from 0.167 to 0.211, are the optimum ratios in satisfying flowability performance and attainment of reasonable compressive strength as high as 80 MPa. It should be emphasised that one of the issues associated with the use of excessive alkali in some conventional alkali-activated formulations is efflorescence, especially for ambient-cured alkali-activated cements [26]. Therefore, the selection of an optimum activator/binder ratio must also consider reducing the amount of activators used so as to increase the economic and environmental benefits of one-part alkali-activated cement.

### 3.3. Test Series C: Water to Binder Ratio

Test series C aimed to investigate the influence of the water/binder (W/B) ratio between 0.40 and 0.55 on the fresh and mechanical properties of the one-part alkali-activated binder. Following the outcomes from test series B, an A/B ratio of 0.08 was selected in the study. The GGBS/binder ratio was kept constant at 0.50 following the results obtained from test series A. A total of four mixes including mixture B3 taken from test series B are listed in Table 8, which summarises the mixture proportions and the molar ratios of critical chemical compounds in the mixtures for this test series.

#### 3.3.1. Fresh Paste Properties

**Figure 11:**
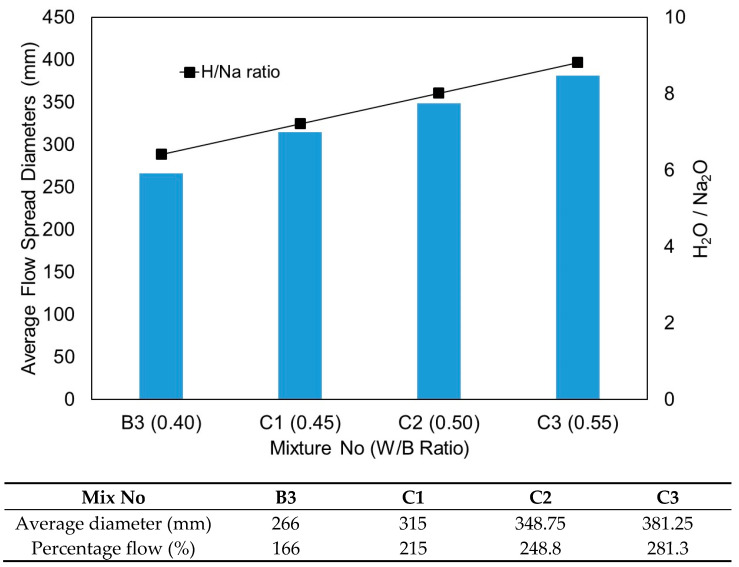
Average flow spread diameters from test series C.

The flow characteristic results from test series C are presented in Figure 11. The influence of the W/B ratio in the one-part alkali-activated binder behaved exactly the same as the water/cement (W/C) ratio in the conventional OPC-based binder. Water content is an important parameter that affects flowability. The increase of the W/B ratio increased the flowability of the mixtures. Another usual way of expressing the amount of water demand in one-part alkali-activated binder studies is the H_2_O/Na_2_O molar ratio [33]. The H_2_O/Na_2_O molar ratios of all mixtures are tabulated in Table 8 and also plotted in Figure 11. As clearly demonstrated in Figure 11, the average flow spread diameters steadily increased with the H_2_O/Na_2_O molar ratios.

#### 3.3.2. Density

**Figure 12:**
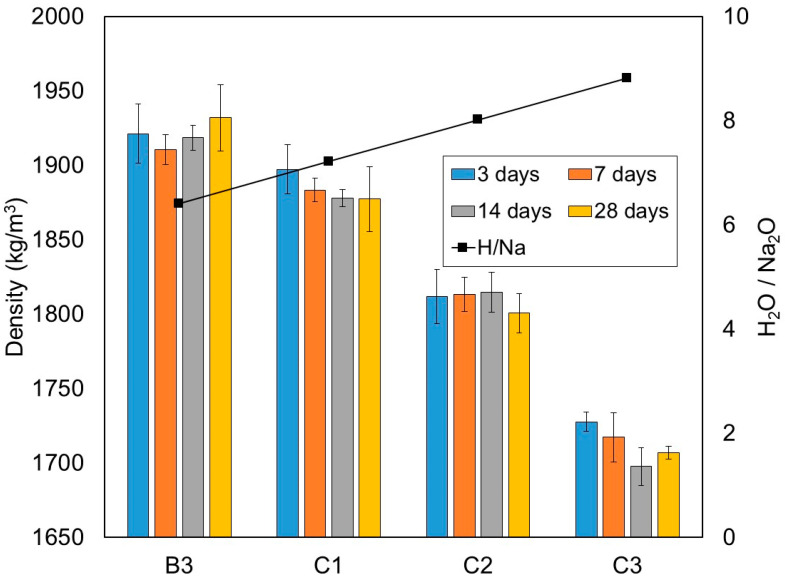
Density of one-part alkali-activated pastes from test series C (kg/m^3^), at the 3, 7, 14 and 28 days of curing periods.

Similar to the previous test series, the density was measured by weighing the mass and dimensions of three 50 mm cubes per age and taking the average value. The dry density results taken from this test series are summarised in Table 9 and Figure 12. Also included in Figure 12 are the curves for the H_2_O/Na_2_O molar ratio. The results show that an increase of the W/B ratio or the H_2_O/Na_2_O molar ratio led to a reduction of density. This was expected as an increase of water content would result in more pores, which leads to reduction of density. As shown in Table 9 and Figure 12, the densities of mixtures C1, C2 and C3 measured at 28 days decreased by 2.83%, 6.8% and 11.65%, respectively, compared to mixture B3.

#### 3.3.3. Compressive Strength

**Figure 13:**
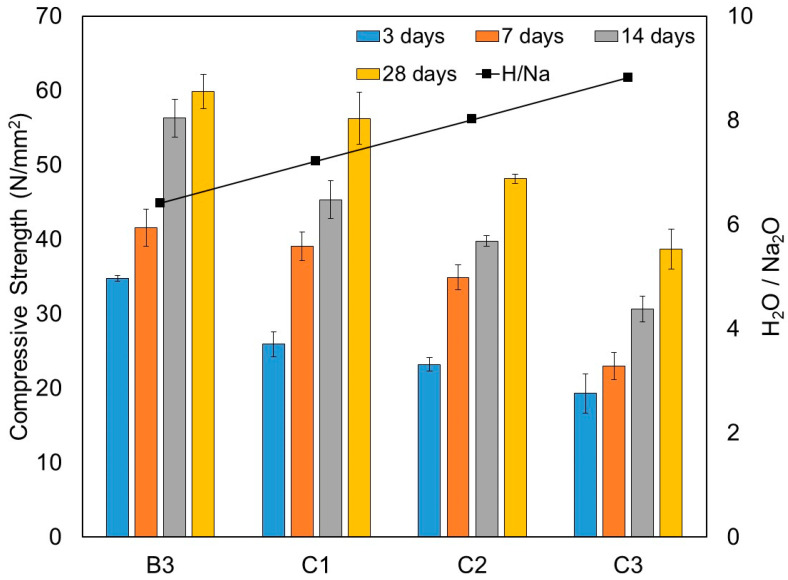
Compressive strength at 3, 7, 14 and 28 days of one-part alkali-activated pastes from test series C (N/mm^2^).

Table 10 summarises the compressive strength results of this series. Similar to dry density, the obtained results showed that the compressive strength decreased with the increase of the W/B ratio or the H_2_O/Na_2_O molar ratio, as clearly shown in Figure 13. The trends consistently agreed throughout all ages of curing in relation to increased water content. In comparison with mixture B3, the compressive strength at 28 days of mixtures C1, C2 and C3 was found to reduce by 6%, 19.6% and 35.4%, respectively.

The strength gain ratios in this test series are calculated and summarised in Table 10. Based on the observation, there was a noticeable reduction in the strength gain ratios with high W/B ratios. This could especially be witnessed between mixtures B3 (W/B = 0.40) and C3 (W/B = 0.55) at ages of 7 and 14 days. Again, this may probably be attributed to the increase in the distribution and size of the pores with the increase in the W/B ratio.

#### 3.3.4. Discussion of Results from Test Series C

The effects of water/binder or H_2_O/Na_2_O molar ratios on the fresh and mechanical properties of one-part alkali-activated binders was investigated in this series. It is well-recognised that water content plays an important role that greatly affects the flowability and strength. With the increase of the water content, the flowability of the mixtures increased, whereas the density and compressive strength decreased.

**Figure 14:**
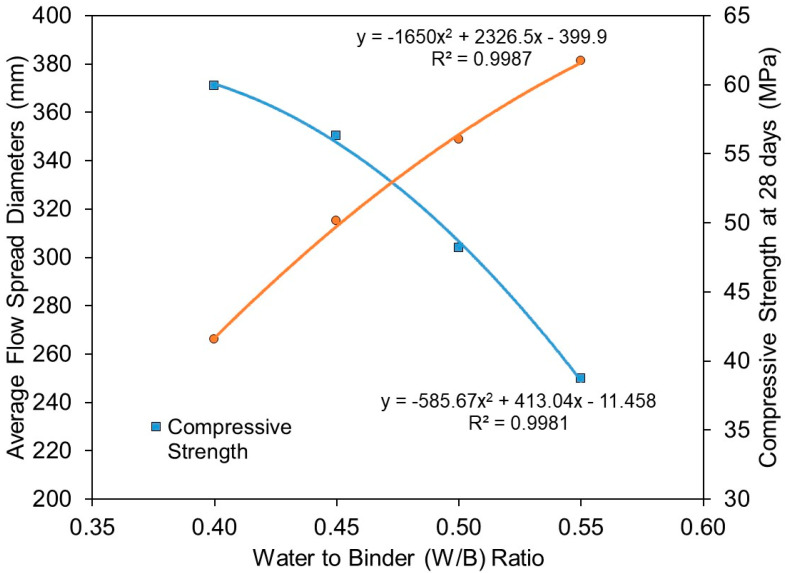
Relationship of average flow spread diameters and compressive strength at 28 days with water to binder ratios (W/B ratio).

The relationship of flowability (in terms of average flow spread diameters) and compressive strength at 28 days with respect to water/binder ratios is plotted in Figure 14. It is evident that the results showed excellent correlations of flowability and compressive strength with water/binder ratios. The flowability performance is directly proportional to the water/binder ratio. On the other hand, compressive strength is inversely proportional to the water/binder ratio. The calculated correlation coefficients (R2) for flowability and compressive strength were 0.9987 and 0.9981, respectively.

As explained by Hajimohammadi et al. [18], water content greatly influenced the dissolution of SiO_2_ from the precursor material and alkali activator. An increase of water content would reduce the alkalinity of the system and lead to lower dissolution of SiO_2_. It is expected that higher alkalinity affects the dissolution of SiO_2_ more than it affects the dissolution of Al_2_O_3_ from source materials. Therefore, more participation of SiO_2_ in stages of gel formation could be observed from mixtures with lower water content. Based on this interpretation, appropriate low water/binder ratios should be considered. So far, our test results suggest that the water/binder ratios should not exceed 0.50 to yield satisfactory performance in flowability and compressive strength.

## 4. Conclusions

The effects of GGBS/binder, activator/binder and water/binder ratios on the fresh and mechanical properties of ambient-cured one-part alkali-activated pastes have been investigated. Combined HCFA and GGBS were used as the precursor materials in this study, with sodium metasilicate anhydrous as the solid alkali activator. The following conclusions can be derived from this study:

1. There was a definite limit on the SiO_2_ content in contributing to compressive strength. Based on our test results, the maximum compressive strength was achieved at the SiO_2_/Al_2_O_3_ molar ratio somewhere between 3.20 and 3.30. Once it passed the limit, the compressive strength decreased.
2. The addition of GGBS with HCFA improved the reactivity of the precursor materials. It enhanced the flowability and compressive strength development in the ambient-cured condition. The optimum GGBS/binder ratio was found at 0.50. It was deduced based on flowability and compressive strength test results from mixture A6, where it achieved a compressive strength of 80 MPa at 28 days with a flow spread diameter of 214.75 mm (114.8% flow).
3. The optimum activator/binder ratio was found in the range between 0.08 and 0.12, which satisfied both the flowability performance (>100% flow) and the attainment of compressive strength up to 80 MPa. There was a limit on the effective amounts of solid activators in the contribution to the alkali-activation reaction process, which was greatly influenced by the SiO_2_ content or the SiO_2_/Al_2_O_3_ molar ratio.
4. Increased water content would increase the flowability, but it reduced the compressive strength and density. Low water/binder ratios should be considered in all cases as this greatly affects the dissolution and participation of SiO_2_ in stages of gel formation. It is recommended that the water/binder ratio should not exceed 0.50.

This paper has summarised findings from a preliminary investigation on the fresh and mechanical properties of one-part alkali-activated binder pastes. The obtained results were shown to be promising. However, the microstructure and morphology analysis was limited and may require further investigation. Future research will focus on the development of tensile strain-hardening characteristics for alkali-activated composites. The effects of different types of aluminosilicate materials and commercially available solid alkali activators on the tensile strain-hardening behaviour of ambient-cured one-part alkali-activated composites will be thoroughly studied in future work.

## Figures and Tables

**Table 1:** Oxide composition of HCFA and GGBS from XRF (by mass).

Material	SiO_2_	Al_2_O_3_	Fe_2_O_3_	CaO	MgO	Na_2_O	K_2_O	TiO_2_	MnO
HCFA (%)	35.07	13.71	13.12	18.94	8.66	4.02	1.18	0.79	0.14
GGBS (%)	32.15	10.60	0.39	43.46	6.58	0.28	0.36	0.65	0.24

**Table 2:** Mixture proportions (by weight) for test series A.

Mix No.	HCFA		GGBS		A/B	Activator	W/B	Water	Molarity			
	%	kg/m^3^	%	kg/m^3^		kg/m^3^		kg/m^3^	Si/Al	Na/Si	H/Na	(Na + K)/Al
A1	100	1153.85	0	0	0.12	138.46	0.40	461.54	2.965	0.25	3.94	0.827
A2	90	1047.14	10	116.35	0.12	139.62	0.40	465.40	3.012	0.242	4.091	0.812
A3	80	938.64	20	234.66	0.12	140.80	0.40	469.32	3.062	0.235	4.254	0.796
A4	70	828.29	30	354.98	0.12	141.99	0.40	473.31	3.113	0.227	4.43	0.780
A5	60	716.05	40	477.37	0.12	143.21	0.40	477.37	3.167	0.219	4.621	0.763
A6	50	601.87	50	601.87	0.12	144.45	0.40	481.49	3.224	0.211	4.83	0.745
A7	40	485.69	60	728.54	0.12	145.71	0.40	485.69	3.284	0.203	5.06	0.726
A8	0	0	100	1258.13	0.12	150.98	0.40	503.25	3.56	0.17	6.238	0.639

HCFA: high-calcium fly ash, GGBS: ground granulated blast furnace slag, A/B: activator/binder ratio, W/B: water/binder ratio, Si/Al: molarity ratio of SiO_2_/Al_2_O_3_, Na/Si: molarity ratio of Na_2_O/SiO_2_, H/Na: molarity ratio of H_2_O/Na_2_O, (Na + K)/Al: molarity ratio of (Na_2_O + K_2_O)/Al_2_O_3_.

**Table 3:** Density of one-part alkali-activated pastes from test series A (kg/m^3^).

Mix No.	3 Days	7 Days	14 Days	28 Days
A1	1906.67	1917.33	1922.67	1916
A2	1950.67	1922.67	1934.67	1921.33
A3	1948	1920	1921.33	1928
A4	1962.67	1949.33	1942.67	1953.33
A5	1958.67	1949.33	1950.67	1948
A6	1972	1961.33	1956	1954.67
A7	1957.33	1958.67	1966.67	1966.67
A8	1952	1961.33	1964	1953.33

**Table 4:** Compressive strength of one-part alkali-activated pastes from test series A.

Mix No.	Compressive Strength (MPa)				Strength Gain Ratio		
	3 Days	7 Days	14 Days	28 Days	f_3_/f_28_	f_7_/f_28_	f_14_/f_28_
A1	0.00	0.00	0.00	0.23	0	0	0
A2	5.81	22.49	41.09	49.49	0.12	0.45	0.83
A3	24.12	46.31	54.89	64.88	0.37	0.71	0.85
A4	39.45	51.64	61.28	73.11	0.54	0.71	0.84
A5	49.24	60.28	67.79	76.65	0.64	0.79	0.88
A6	57.09	64.07	68.63	80.07	0.71	0.80	0.86
A7	57.43	67.68	74.51	90.88	0.63	0.74	0.82
A8	49.92	70.07	77.88	78.45	0.64	0.89	0.99

f_3_: Compressive strength at 3 days, f_7_: compressive strength at 7 days, f_14_: compressive strength at 14 days, f_28_: compressive strength at 28 days.

**Table 5:** Mixture proportions (by weight) for test series B.

Mix No.	HCFA		GGBS		A/B	Activator	W/B	Water	Molarity			
	%	kg/m^3^	%	kg/m^3^		kg/m^3^		kg/m^3^	Si/Al	Na/Si	H/Na	(Na + K)/Al
B1	50	627.03	50	627.03	0.04	50.16	0.40	501.62	2.918	0.118	9.54	0.408
B2	50	620.54	50	620.54	0.06	74.47	0.40	496.43	2.995	0.143	7.67	0.492
B3	50	614.19	50	614.19	0.08	98.27	0.40	491.35	3.071	0.167	6.41	0.577
B4	50	607.97	50	607.97	0.10	121.59	0.40	486.37	3.148	0.19	5.51	0.661
A6	50	601.87	50	601.87	0.12	144.45	0.40	481.49	3.224	0.211	4.83	0.745
B5	50	595.89	50	595.89	0.14	166.85	0.40	476.71	3.301	0.232	4.30	0.829

HCFA: high-calcium fly ash, GGBS: ground granulated blast furnace slag, A/B: solid activator/binder ratio, W/B: water/binder ratio, Si/Al: molarity ratio of SiO_2_/Al_2_O_3_, Na/Si: molarity ratio of Na_2_O/SiO_2_, H/Na: molarity ratio of H_2_O/Na_2_O, (Na + K)/Al: molarity ratio of (Na_2_O + K_2_O)/Al_2_O_3_.

**Table 6:** Density of one-part alkali-activated pastes from test series B (kg/m^3^).

Mix No.	3-Day	7-Day	14-Day	28-Day
B1	1925.33	1885.33	1880	1868
B2	1898.67	1910.67	1894.67	1906.67
B3	1921.33	1910.67	1918.67	1932
B4	1966.67	1942.67	1948	1942.67
A6	1972	1961.33	1956	1954.67
B5	1976 *	1960 *	1964 *	1966

* The result is based on only one cube specimen due to damage of cube specimens during the demoulding stage.

**Table 7:** Compressive strength of one-part alkali-activated pastes from test series B (N/mm^2^).

Mix No.	Compressive Strength (MPa)				Strength Gain Ratio		
	3 Days	7 Days	14 Days	28 Days	f_3_/f_28_	f_7_/f_28_	f_14_/f_28_
B1	16.27	22.89	27.68	31.09	0.52	0.74	0.89
B2	25.80	34.51	40.39	47.04	0.55	0.73	0.86
B3	34.79	41.60	56.31	59.89	0.58	0.69	0.94
B4	40.73	53.57	64.83	70.81	0.58	0.76	0.92
A6	57.09	64.07	68.63	80.07	0.71	0.80	0.86
B5	55.60 *	66.48 *	78.24 *	79.86	0.70	0.83	0.98

* The result is based on only one cube specimen due to damage of cube specimens during the demoulding stage. f_3_: Compressive strength at 3 days, f_7_: compressive strength at 7 days, f_14_: compressive strength at 14 days, f_28_: compressive strength at 28 days.

**Table 8:** Mixture proportions (by weight) in test series C.

Mix No.	HCFA		GGBS		A/B	Activator	W/B	Water	Molarity			
	%	kg/m^3^	%	kg/m^3^		kg/m^3^		kg/m^3^	Si/Al	Na/Si	H/Na	(Na + K)/Al
B3	50	614.19	50	614.19	0.08	98.27	0.40	491.35	3.071	0.167	6.412	0.577
C1	50	578.65	50	578.65	0.08	92.58	0.45	520.78			7.214	
C2	50	547	50	547	0.08	87.52	0.50	547			8.015	
C3	50	518.63	50	518.63	0.08	82.98	0.55	570.49			8.817	

HCFA: high-calcium fly ash, GGBS: ground granulated blast furnace slag, A/B: solid activator/binder ratio, W/B: water/binder ratio, Si/Al: molarity ratio of SiO_2_/Al_2_O_3_, Na/Si: molarity ratio of Na_2_O/SiO_2_, H/Na: molarity ratio of H_2_O/Na_2_O, (Na + K)/Al: molarity ratio of (Na_2_O + K_2_O)/Al_2_O_3_.

**Table 9:** Density of one-part alkali-activated pastes from test series C (kg/m^3^).

Mix No.	3 Days	7 Days	14 Days	28 Days
B3	1921.33	1910.67	1918.67	1932
C1	1897.33	1883.47	1878.13	1877.33
C2	1812	1813.33	1814.93	1800.8
C3	1727.73	1717.33	1697.87	1706.93

**Table 10:** Compressive strength of one-part alkali-activated pastes from test series C (MPa).

Mix No.	Compressive Strength (MPa)				Strength Gain Ratio		
	3 Days	7 Days	14 Days	28 Days	f_3_/f_28_	f_7_/f_28_	f_14_/f_28_
B3	34.79	41.60	56.31	59.89	0.58	0.69	0.94
C1	25.92	39.08	45.36	56.29	0.46	0.69	0.81
C2	23.21	34.92	39.81	48.17	0.48	0.72	0.83
C3	19.31	23	30.67	38.71	0.50	0.59	0.79

f_3_: Compressive strength at 3 days, f_7_: compressive strength at 7 days, f_14_: compressive strength at 14 days, f_28_: compressive strength at 28 days.

## Data Availability

Not applicable.
